# Effect of Dexmedetomidine combined with sufentanil for post- thoracotomy intravenous analgesia:a randomized, controlled clinical study

**DOI:** 10.1186/s12871-017-0324-4

**Published:** 2017-03-01

**Authors:** Chun-Shan Dong, Jun Zhang, Qiang Lu, Peng Sun, Jun-Ma Yu, Chao Wu, Hao Sun

**Affiliations:** Department of Anesthesiology, Third affiliation hospital of Anhui Medical University, Hefei huaihe road No. 390, Hefei, 230061 China

**Keywords:** Post-thoracotomy, Sufentanil, Dexmedetomidine, Combination, Intravenous analgesia

## Abstract

**Background:**

Few studies have investigated the use of dexmedetomidine in patient-controlled intravenous analgesia (PCIA) after thoracic surgery. This study to evaluate the effect of dexmedetomidine combined with sufentanil for PCIA after thoracotomy under general anaesthesia.

**Methods:**

Ninety-seven adults patients scheduled for thoracotomy surgery. All two groups received PCIA with either sufentanil alone (control group) or combining dexmedetomidine with sufentanil (dexmedetomidine group). Hemodynamic measurements, visual analog scale (VAS) scores at rest and at coughing, Ramsay sedation score (RSS), analgesic consumption, and postoperative nausea and vomiting (PONV) as well as drug-related adverse effects were compared at 2, 6, 12, 24, 36 and 48 h postoperatively.

**Results:**

In the patients of the dexmedetomidine group, compared to the control group, the pain scores at rest or at coughing during 48 h postoperatively were lower (*P* < 0.001), the sedation scores were lower, the consumption of sufentanil and rescue meperidine were lower, and the number of episode of moderate PONV was three times lower. No signs of toxicity or local complications were observed. There was a non-significant trend for a lower HR and BP in the dexmedetomidine group vs. Control.

**Conclusion:**

The combining dexmedetomidine with sufentanil for post-thoracotomy PCIA can improve pain control together with the decrease in sufentanil requirements, and improve postoperative patient’s satisfaction compared with sufentanil alone in PCIA.

**Trial Registration:**

This trial was retrospectively registered on 27 April 2016 at the Chinese Clinical Trial Register (number: ChiCTR-ONC-16008376).

## Background

Patients undergoing thoracic surgery experience severe pain and to have supreme effect on painful respiratory movements in the postoperative period, in whom it is reflected by the long duration of patient-controlled analgesia (PCA), compromised pulmonary function can occur when uncertain analgesia, and high morphine consumption [1, 2]. The use of morphine is associated with side effects, which the most important side effects include nausea, vomiting, sedation and respiratory depression during acute morphine therapy [3]. Therefore, one of the most promising interventions requiring investigation is the reduction of postoperative opioid consumption and pain intensity by an adjunct drugs with an opioid, such as α2-adrenoceptor agonists.

Some studies report that perioperative and postoperative systemic usage of dexmedetomidine provides considerably more satisfactory analgesic effect, reduces PCA morphine requirements and lower incidence of postoperative side effects compared to morphine alone [4–6]. Compared with morphine, sufentanil improve immediate postoperative pain control with a strong analgesic effect; however, because patients undergoing complex thoracotomy may be continue to experience respiratory depression and increase the incidence of other complications, an adjunct drug such as dexmedetomidine, has been suggested as a safe alternative to together with sufentanil by way of patient-controlled intravenous analgesia (PCIA) [7]. However, the beneficial properties of dexmedetomidine has been described as involving analgesia, anxiolysis, sedation and sympatholysis, the neural networks involving the peripheral and central mechanisms has been hypothesized to play a major role in determining less likelihood of adverse-effect profile and opioid-sparing effect during patient-controlled intravenous analgesia (PCIA) [8–10].

Our goal was to compare the pain intensity, analgesics consumption, hemodynamic and the drug-related adverse effect of PCIA with combining dexmedetomidine with sufentanil or sufentanil alone protocol in a prospective randomized study design after thoracotomy operations.

## Methods

### Ethics approval and consent to participate

This randomized, double-blind, and single-centre study enrolled sixty-five patients from April 2014 to June 2015. The study was approved by the ethics committee of the third affiliated hospital of AnHui Medical University for human studies (Ethical Committee number HFYY2014002) on January 2014, and written informed consent was obtained from all patients (Chinese Clinical Trial number, ChiCTR-ONC-16008376). This study protocol complied with the 1975 Declaration of Helsinki.

### Criteria for inclusion and exclusion

Patients who were classified as American Society of Anesthesiologists (ASA) physical status I to II, aged between 32–65 years and scheduled to undergo elective major open thoracotomy operation under general anesthesia were recruited. Patients were excluded with a serious central nervous system pathology, a left ventricular ejection fraction of <35%, greater than first-degree atrioventricular block and rate-controlled atrial fibrillation, acute or chronic hepatitis, a requirement for renal supplementation, a known uncontrolled seizure disorder, use of psychiatric medications, drug abuse other than alcohol, or cognitive impairment or if they were pregnant or lactating.

### Preoperative preparations and anesthesia protocol

Prior to enrollment, patients were screened for study eligibility. Besides, the patients who refused to fill out the informed consent was excluded from study. A resident of anesthetist visited the patients before the operation and described the visual analogue scale (VAS) for them. Meanwhile, he prescribed the premedication and 8 h nil-per-os for the patients. All of the patients were anesthetized by an anesthetist who was blinded to study and didn’t participate in data collection. Furthermore, the anesthetist resident who collected post-operative data were blinded to study and this process continued to end of the study. All the patients were fully informed about the study and blinded to their groups. Before surgery, patients were instructed on the operational use of PCIA bump and a 0–10 VAS measurements of pain.

All patients were pre-medicated with midazolam 0.05 mg.kg^−1^ was IV injected 2 h before surgery and received IV 500 ml of acetate Ringer’s sodium over 30 min before induction of anesthesia. Upon arrival in operating room usual monitoring [including ECG, pulse oxygen saturation (SpO_2_), noninvasive blood pressure (BP)] was established, and the hemodynamic measurements in the operating room were recorded as a preoperative baseline values. Narcotrend monitor (Narcotrend index: NI) were applied before the induction of anesthesia in order to obtain the anesthesia depth levels. All patients received 0.4 μg.kg^−1^ sufentanil (sufentanil Citrate; Inc., RenFu Pharmaceutical, China), and propofol, sevoflurane and cisatracurium for induction and tracheal intubation, and then maintenance of anesthesia with a target concentration infusion of sufentanil (effect-site concentration) 1.0 μg.kg^−1^.h^−1^ for intra-operative analgesia, followed by maintenance with propofol, sevoflurane, oxygen, and cisatracurium. Intra-operation, a variable concentration infusion regimen of propofol and sevoflurane were adjusted as necessary to maintain an NI values between 40 to 50 as well as haemodynamic responses to surgical stimuli. Edrophonium and atropine were given to reverse residual neuromuscular block at the end of surgery. Patients emerged from the operating room extubated and total recovery from anesthesia with stayed 1 h in the post anesthetic care unit (PACU) (as judged by the ability to obey verbal commands on request and stable hemodynamic variables). All patients were attached to an electronic infusion pump (JA5806 PCA; Inc., ShangHai ANGEL, ED, China) for PCIA and being fully awake allowed to back the general ward. The study commenced in the postoperative period.

### Postoperative PCIA strategy

A standard sufentanil in PCIA protocol was adopted throughout the studied period. According to the randomization plan, the PCIA regimen consisted of sufentanil 3.0 μg.kg^−1^ and 8 mg ondansetron, mixed with 0.9% normal saline to a total volume of 250 ml. In the dexmedetomidine group, in addition to the sufentanil and ondansetron, 4.0 μg.kg^−1^ of dexmedetomidine (Precedex; Aibeinin^®^, Inc., Henrui Pharmaceutical, China) was added to the PCIA solution and again the volume was made up to 250 ml with 0.9% normal saline. The PCIA was programmed to deliver a 2 ml bolus on-demand, with a lock-out interval of 10 min, and a background infusion rate of 4 ml.h^−1^. All patients received 20 ml i.v. of PCIA solution immediately after were attached a PCIA pump. During the study period after surgery, this PCIA programme thus allowed a continuous background infusion of sufentanil 0.048 μg.kg^−1^.h^−1^ and a bolus of sufentanil 0.024 μg.kg^−1^; and allowed a continuous background infusion of dexmedetomidine 0.064 μg.kg^−1^.h^−1^ and a bolus of dexmedetomidine 0.032 μg.kg^−1^. The PCIA was used for the first 48 h postoperatively. Upon arrival in the general ward, all the patients were once again instructed on the use of the PCIA pump and VAS shortly, and they were encouraged to push the PCIA button (self-administer their own PCA medications) to achieve an rescue analgesic when their could not tolerance pain throughout 48 h after operation.

### Outcome measures and data collection

The study outcomes and the vital parameters were recorded at 2, 6, 12, 24, 36 and 48 h after arrival to the ward. During the studied period, BP, HR and SpO_2_ were monitored and were recorded at above time points. Postoperative analgesia was used through the PCIA pump over 48 h. Pain intensity was evaluated with VAS at rest (VASR) and at coughing (VASC). VASR was assessed with the patient lying supine and VASC was assessed during change from coughing. The VAS scores from zero (pain free) to 10 (maximum level of pain). The restlessness scores was estimated based on Ramsay sedation scale (RSS) from 1 to 6 (1 = anxious and agitated, 2 = cooperative, tranquil, oriented, 3 = responds only to verbal commands, 4 = asleep with brisk response to light stimulation, 5 = asleep without response to light stimulation, 6 = non responsive) [11]. Nausea and vomiting (PONV) scores from 1 to 4 (1 = without nausea and vomiting, 2 = nausea without vomiting, 3 = less than two times vomiting, 4 = severe vomiting more than two times) and satisfaction scored from 1 to 4 (1 = not satisfied, 2 = moderately satisfied, 3 = satisfied, 4 = very satisfied) [12, 13]. The patients were encouraged self-administer their own PCIA medications to achieve an rescue analgesic if the patient with pain scores more than 3 at rest or during cough, the setting for PCIA was one bolus with 10 min lockout. If patients required more than three self-administer of rescue medications for pain within 1 h, an adjuvant analgesic with IV injection meperidine 50 mg would be administered for insufficient analgesia. The dosage of meperidine could be repeated per 1 h and after each meperidine administration. The number of PCIA self-administer and meperidine injection were recorded. Persistent nausea, vomiting, or pruritus would warrant PCIA termination with the patient then being switched to an alternate analgesia modality. Nausea and vomiting episodes were assessed at the same intervals and recorded as present, with regard to the severe PONV, when all patient were asked having degree scores ≥ 3 at each time interval were counted. The drug-related bradycardia (HR < 60 beats/min), hypotension (>20% decrease in systolic blood pressure (SBP) or > 15% decrease in diastolic blood pressure (DBP) from preoperative baseline), RSS≧3, and respiratory depression (ventilatory frequency < 8 beats per minute (bpm) lasting for more than 10 min) were considered as severe adverse events. If severe adverse events occurred, the use of PCIA was stopped temporarily and patient was observed continuously for 30 min. If the adverse effects appear more than 30 min or a further severe, the patient were treated with appropriate medications. Hypotension or bradycardia was treated with volume expansion, ephedrine, or atropine. Respiratory depression was treated with naloxone and oxygen. Nausea and vomiting were treated with metoclopramide. The presence of pruritus was recorded and treated with diphenhydramine. Each patient was asked to grade satisfaction (described above from 1 to 4) with pain and sedation situation at the of PCIA use.

### Statistical analysis

To properly power this study, power analysis for testing the hypothesis and determine sample size used a primary endpoint defined as sufentanil consumption at 48. In the study literature. [6], the mean ± SD morphine consumption for 48 h with and without dexmedetomidine were 35 ± 28 and 54 ± 37 mg, respectively, a 41% reduction of morphine use with dexmedetomidine. In our study, the hypothesis was that compared with control group, dexmedetomidine group would achieve a 30% reduction of sufentanil consumption. A sample size of 25 patients was calculated using StatMate (ver.2.0; GraphPad Software, San Diego, CA) to have at least 80% power with α value of 0.05 significance level (two-tailed). Taking exclusion into account, we aimed to recruit 30 patients in each group.

Statistical analysis was performed using SPSS 13.0 (SPSS Inc., Chicago, USA). To compare patient characteristics and operative data between the groups, independent t-tests or Mann–Whitney U tests for continuous variables, and Fischer exact tests or Chi-square tests for categorical variable were performed. To compare changes in VAS scores, Ramsay sedation scores, and the PONV scores as well as hemodynamic variables between the groups, a general linear model used followed by two-way analysis of variance (ANOVA) using *post hoc* testing for repeated measures. The cumulative PCIA analgesic consumption and numbers of rescue analgesic were analyzed by Pair-simple tests as appropriate. The differences in the proportions of PONV severity and adverse events were analyzed by Fisher exact tests or Chi-square tests as appropriate. Overall satisfaction was compared between the groups using the Kruskal-Wallis H test. All results were expressed with 95% confidence interval (CI), number of patients (%), mean rank or odds ratio with 95% CI. A probability level of < 0.05 was considered to be statistically significant.

## Results

**Fig. 1:**
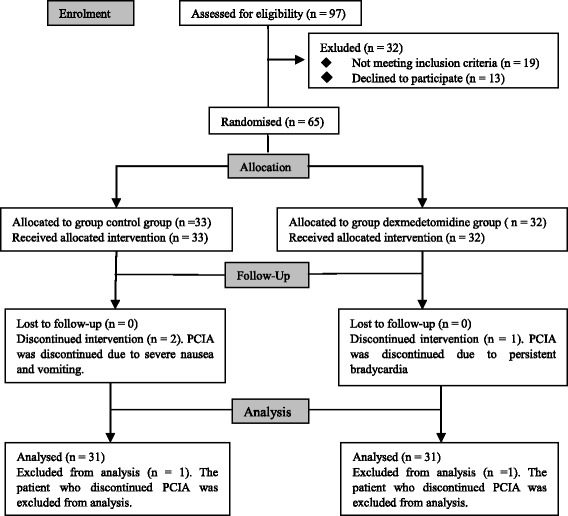
Patients enrollment flow diagram. PCIA: patient-controlled intravenous analgesia

Sixty patients completed the study: 30 in each group, a flowchart is shown in Fig. 1. There were no significant differences between the groups regard to patient characteristics and intra-operative variables (Table 1).

**Table 1:** Patient characteristics and intraoperative data. Values are mean ± SD, or number. All variable were similar between the two groups

	control group (*n* = 30)	Dexmedetomidine group (*n* = 30)	p
Age (years)	58 ± 8	56 ± 8	0.533
Weight (kg)	67 ± 7	68 ± 8	0.562
Height (cm)	167 ± 7	168 ± 7	0.551
Body mass index (kg.m2)	24 ± 1	24 ± 1	0.642
Sex (male/female)	4/16	15/15	0.796
ASA physical status (I/II)	5/25	2/28	0.421
Length of anesthesia (min)	137 ± 50	142 ± 47	0.638
Intraoperative sufentanil consumption (mcg)	70 ± 20	73 ± 24	0.535
Type of surgery (n)			
Esophageal neoplasia resection	16	17	0.795
Lobectomy	6	7	0.754
Pneumonectomy	1	2	1.000
Mediastinal mass	2	2	NA
Pneumothorax	5	2	0.421
Extubated in operating room (number of patients)	21	1	0.584

Abbreaviations: *SD*, standard deviation; *ASA*, american society of anesthesiologists physical status

**Fig. 2:**
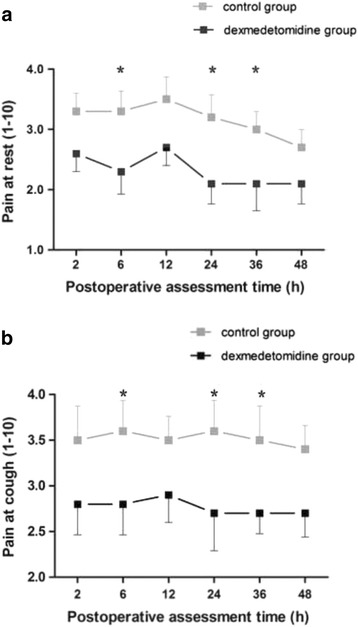
Postoperative visual analogue scale (VAS) pain scores. Evaluation of pain score on VASR **(a)** and VASC **(b)** (Values represent means with 95% confidence interval) in the two groups. *P* < 0.001 by General Linear Model analysis, subsequent comparison between the groups: asterisk = *P* < 0.023 in VASR and *P* < 0.02 in VASC after the *post hoc* analyses, dexmedetomidine group vs. control group

**Fig. 3:**
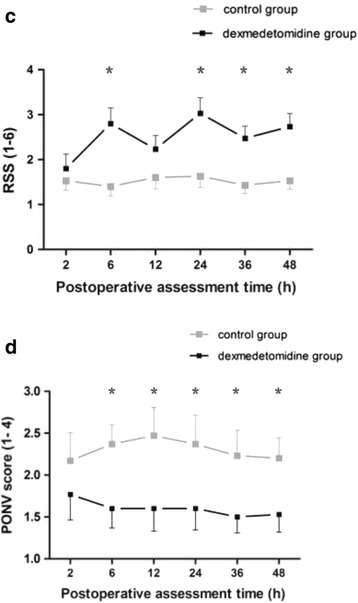
Postoperative Ramsay sedation scale (RSS). **(a)** restlessness scores and PONV scores. **(b)** Evaluation of RSS and PONV scores (Values represent mean with 95% confidence interval) in the two groups. *P* < 0.001 by General Linear Model analysis, subsequent comparison between the groups: asterisk = *P* < 0.05 after the *post hoc* analyses, dexmedetomidine group vs. control group. PONV, postoperative nausea and vomiting

There was a significant difference in VASR and VASC between the groups (*P* < 0.001). Individual intergroup comparison at each time point are shown in Fig. 2 *.* (a) and (b). RSS scores was lower in the dexmedetomidine group than in the control group (Fig. 3. (a). *P* < 0.035).

**Fig. 4:**
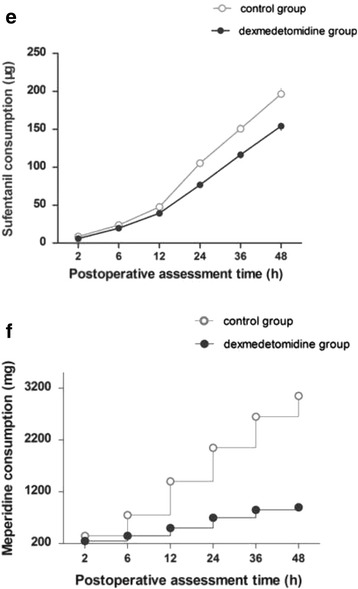
Cumulative PCIA sufentanil consumption and supplementary meperidine doses. **(a)** There is significantly differences in cumulative PCIA sufentanil consumption at 48 h between in the control group and the dexmedetomidine group (Values represent means with 95% confidence interval, cumulative dose at 48 h for 196.6 ± 22.2 vs. 154.3 ± 17.5 μg, *P* = 0.034). Sufentanil consumption in the dexmedetomidine group was required 22% less than the control group. **(b)** Cumulative of meperidine supplementary doses at each time intervals after surgery was significantly lesser in the dexmedetomidine gourp than the control group (*P* = 0.002). PCIA, patient-controlled intravenous analgesia; SD, standard deviation

Pair-simple comparisons (mean) showed a lower sufentanil consumption in the dexmedetomidine group than in the control group at 48 h postoperatively (Fig. 4. (a)). Total mean doses for each patient: 154 vs. 196 μg/sufentanil, *P* = 0.034). Meperidine requirement were statistically different between the groups over time (Fig. 4. (b). *P* = 0.002).

**Fig. 5:**
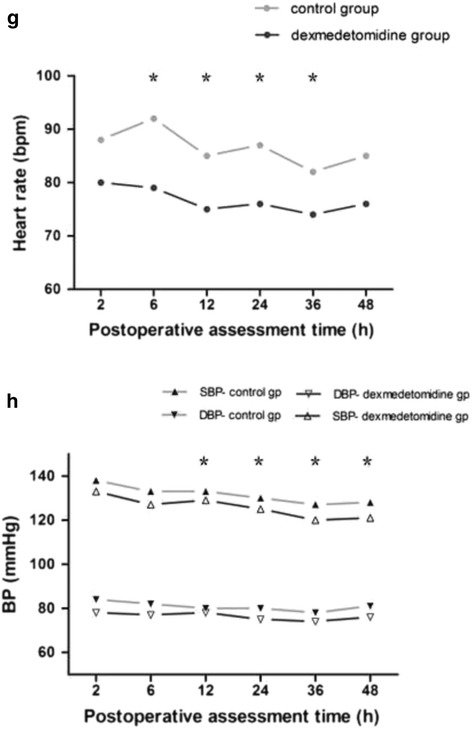
Postoperative hemodynamic variables was assessed at 2, 6, 12, 24, 36 and 48 h postoperatively (Values represent means with 95% confidence interval). Although the heart rate **(a)**, systolic pressure and diastolic pressure **(b)** were compared between the groups postoperatively. asterisk = *P* < 0.05 by the *post hoc* analyses after a General Linear Model, dexmedetomidine group vs. control group

**Fig. 6:**
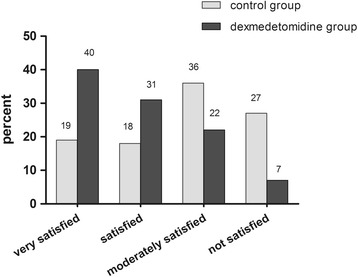
Patients’ overall postoperative satisfaction rating with 48 h (control group vs dexmedetomidine group). The satisfaction score was assessed using a 4-point scale (not satisfied, moderately satisfied, satisfied, very satisfied). The four categories (*N* = 180, Mean Rank was 146.3 vs 214.7, *x* ^2^ = 41.8, *df* = 1, *P* = 0.000)

With regard to the PONV (Fig. 3. (b)), comparison of the risk estimate by PONV scores ≥ 3, patients in the dexmedetomidine group had a significantly reduced risk of PONV during the 6 to 48 h compared with control group (odds ratio 0.184, 95% CI 0.09 to 0.38; *P* < 0.001), and the incidences of PONV between the groups during 48 h was comparable (12% vs 33%). No difference was found in the occurrence of other complications, such as haemodynamic or respiratory. No life-threatening complication related to the use of the two protocol occurred (Table 2). Although there are statistically differences in HR or BP values at some time points (Fig. 5. (a) and (b)), there was a non-significant trend for a lower HR and BP in the dexmedetomidine group vs. Control. Accordingly, the overall postoperative satisfaction rating for 48 h were different between the two groups (*n* = 180, Mean Rank was 146.3 vs 214.7, *x* ^2^ = 41.8, *df* = 1, *P* = 0.000) (Fig. 6).

**Table 2:** The comparison of postoperative complications between two goups

	control group (*n* = 30)	Dexmedetomidine group (*n* = 30)	*P*
Postoperative complications, No %			
Hypotension	0 (0)	2 (6.7)	0.472
Hypertension	7 (23.3)	4 (13.3)	0.505
Bradycardia	2 (6.7)	7 (23.3)	0.148
Respiration despression	9 (30.0)	4 (13.3)	0.117
Hypoxemia	6 (20.0)	142 ± 47	1.000
Excessive sedation	2 (6.7)	73 ± 24	0.083
Pruritus	7 (23.3)	2 (6.7)	0.147

Note: Hypotension, hypertension, bradycardia, respiration depression, hypoxemia and excessive sedation were defined as mean blood pressure < 60 mmHg, or > 90 mmHg, heart rate < 60 beat per minute, respiratory rate < 8 beat per minute, pulse oxygen saturation < 93%, and Ramsay sedation scale ≥ 3 at least once during postopeartive 48 h, respectively. No, number

## Discussion

We have demonstrated that intense postoperative pain after thoracotomy can be treated successfully with a lower dose continuous infusion of combining dexmedetomidine with sufentanil in PCIA. The combination of dexmedetomidine and sufentanil allowed a significant reduction in sufentanil consumption, supplemental analgesic requirements and decreased incidence of nausea or vomiting, and to maintain a good hemodynamic stability.

Increased levels of safety and efficacy of PCIA protocol are desirable for patients who have undergoing a major thoracotomy, while protecting the pulmonary function and promotes patient well-being. However, a numberous strategies have been designed to reduce postoperative pain for various surgery, and dexmedetomidine as an adjunct combining with an opioid in PCIA for pain managements has been reported to be associated with maximizing pain relief and minimizing analgesic-related side-effects [5]. From the clinical viewpoint, however, the drug doses for patients can be calculated simply on per kg basis is importance (except for obese patients). The recommended infusion dose of dexmedetomidine is 0.2 to 0.7 μg.kg^−1^.h^−1^ for a major abdominal surgery in adults, and showed a reduction in ‘rescue’ opioid consumption in the first 24 h after surgery, together with in general no clinically important differences in postoperative pain when compared with placebo [14]. In addition, a similar results were found in the context of thoracic surgery, an infusion dose of 0.04 μg.kg^−1^.h^−1^ dexmedetomidine combining with 0.02 μg.kg^−1^.h^−1^ sufentanil reduced postoperative pain and greater patient satisfaction without other clinically relevant side effect for highly nicotine-dependence patients during the initial 72 h after surgery [15, 16]. We selected a maintenance infusion of 0.064 μg.kg^−1^.h^−1^, the maintenance time of dexmedetomidine consumed in conjunction with sufentanil via PCIA was 48 h of post-thoracotomy analgesic, which almost is negligible among the lowest limit used for sedation within the ranges of the recommended infusion. This may explain why receiving dexmedetomidine required 22% less PCIA sufentani compared with sufentanil alone, such as analgesic and sedation, are affected differently by dexmedetomidine doses [17]. while we have also found that beneficial influence on optimal balance between postoperative pain and side effects with the time period when cumulative sufentanil consumption was significantly lower in the dexmedetomidine group. Obviously, these advantages shown by the regime is very important for thoracic surgery during the initial two day with a consequent decrease in lung volumes and capacities due to the pain response [18].

The sedation measures scores in the dexmedetomidine group were significantly better than the control group at all time points; however, sedation and analgesia probably account for the opioid-sparing effects of this class of compound [19]. We suggest that some more serious complications such as PONV and wound dehiscence in the postoperative context after major elective surgery might be benefited by the use of dexmedetomidine due to an appropriate sedative effect [20, 21]. For these patients, an opioid-based PCIA regimen may also provide additional physiological benefits by a multimodal approach that exert opioid-sparing effect as a feasible option. However, sufentanil, although induces side effects characteristic of all opioids, even advanced sedation levels precede respiratory depression, it remain attributed to excessive dosing as a factor [22, 23]. A wider therapeutic index in sufentanil has been demonstrated compared with other commonly used opioids for PCIA, and sufentanil is not processed into active metabolites [24, 25]. Also, the optimal dose of dexmedetomidine combining with sufentanil is vital and clinically the analgesic and sedation is satisfied maximumly, and the consumption of sufentanil is reported to be reduced when administered together with dexmedetomidine [26]. A recent study *in vivo* have showed that a lower loading dose of dexmedetomidine (<10 μg/kg) prevent response to noxious stimulation mainly via enhanced inhibitory postsynaptic transmission within the superficial dorsal horn without altering excitatory synaptic transmission or evoking direct postsynaptic membrane currents. In contrast, higher doses of dexmedetomidine (>10 μg/kg) induced outward currents by a direct postsynaptic action [27]. Therefore, experimental data suggest dexmedetomidine synergistic analgesic interactions with opioids on the cortex at low concentrations, which is consistent with our observation at the concentration range of opioid-sparing effects. A strong linear relation between an area under the dexmedetomidine concentration-time curve and a cumulative dexmedetomidine dose has been demonstrated in critically ill patients [28]. This has led to the hypothesis that opioid-sparing effects of dexmedetomidine was produced by inhibits pain transmission with a concentration dependent in the peripheral and central.

The other most important to understand what was induced through pain response for the interpretation of our result. Because of the major surgical procedure and long anaesthesia are also main sources of stress to patients. During surgery, the suppression of inflammation to painful stimuli is an important component of analgesia. Tissue injury are able to stimulate the release of cytokines and chemokines in cell signaling events underlying inflammation, and excessive release of cytokines and chemokines has been reported to be associated with pain associated with tissue injury. Several studies in human have shown that the excessive activation of the sympathetic nervous system and inflammation were alleviated by central sympatholytic effects of dexmedetomidine during the cytokine secretion secondary to immune system interactions [29, 30]. Therefore, the likely involvement of the inhibition of nuclear factor kappa-*β* activation in the mechanism of dexmedetomidine action suggeats that the nuclear factor kappa-*β* receptor antagonists may beneficially inhibit inflammatory responses associated with organ injury and acute or chronic pain conditions [31].

Our study has some limitation. First, there are limited large sample data on perioperative outcome comparisons of patients undergoing thoracotomy procedure who had either a opioids or dexmedetomidine as an aduvants in PCIA for postoperative pain management. Second, dexmedetomidine was administered at a alone individual infusion rate of 0.064 μg.kg^−1^.h^−1^ for postoperative analgesia. We did not measure the serum concentration of dexmedetomidine in this study at any time point. Third, we only had investigated middle-aged adults patients after thoracic surgery, so it remains to be proven whether our finding are also applicable to young and elderly adults patients after thoracic surgery or patients undergoing other types of surgery. Finally, this study was performed at a single center. Investigation of more diverse populations from different center and using various techniques would furnish more conclusive results. Again, despite the potential benefit of dexmedetomidine include a significant opioid-sparing effects for PCIA, it is not labelled for such use so a current use is not recommendable yet.

## Conclusions

Our results show the benefit of dexmedetomidine (0.064 μg.kg^−1^.h^−1^) combined with sufentanil (0.048 μg.kg^−1^.h^−1^) in PCIA regarding post-thoracotomy pain and sufentanil requirements, and more particularly in greater overall patient satisfaction by a appropriate sedation and reduced the incidence of nausea or vomiting with PCIA. These finding are in agreement with those of the literature, and more studies are still required to determine the optimal dose of dexmedetomidine for reducing postoperative pain and sufentanil requirements in young or elderly adult patients after thoracic surgery.

## Abbreviations

ASA: American society of anesthesiologists status
BP: Blood pressure
bpm: Beats per minute
DBP: Diastolic blood pressure
HR: Heart rate
i.v: Intravenous infusion
PACU: Post-anesthetic care unit
PCA: Patient-controlled analgesia
PCIA: Patient-controlled intravenous analgesia
PONV: Postoperative nausea and vomiting
RSS: Ramsay sedation scores
SBP: Systolic blood pressure
SpO_2_: Pulse oxygen saturation
VAS: Visual analogue scores
VASC: Visual analogue scores at coughing
VASR: Visual analogue scores at rest
